# OR2AT4, an Ectopic Olfactory Receptor, Suppresses Oxidative Stress-Induced Senescence in Human Keratinocytes

**DOI:** 10.3390/antiox11112180

**Published:** 2022-11-03

**Authors:** Ji-Sun Kim, Ha Lim Lee, Ji Hyun Jeong, Ye Eun Yoon, In-Ryeong Lee, Ji Min Kim, Chunyan Wu, Sung-Joon Lee

**Affiliations:** Department of Biotechnology, Graduate School of Life Science & Biotechnology, College of Life Science and Biotechnology, Korea University, Seoul 02841, Korea

**Keywords:** olfactory receptor, OR2AT4, sandalore, senescence, human keratinocytes, CaMKKβ/AMPK/mTORC1/autophagy signaling

## Abstract

Olfactory receptors (ORs) are the largest protein superfamily in mammals. Certain ORs are ectopically expressed in extranasal tissues and regulate cell type-specific signal transduction pathways. OR2AT4 is ectopically expressed in skin cells and promotes wound healing and hair growth. As the capacities of wound healing and hair growth decline with aging, we investigated the role of OR2AT4 in the aging and senescence of human keratinocytes. OR2AT4 was functionally expressed in human keratinocytes (HaCaT) and exhibited co-expression with G-protein-coupled receptor signaling components, G_olfα_ and adenylate cyclase 3. The OR2AT4 ligand sandalore modulates the intracellular calcium, inositol phosphate, and cyclic adenosine monophosphate (cAMP) levels. The increased calcium level induced by sandalore was attenuated in cells with *OR2AT4* knockdown. OR2AT4 activation by sandalore inhibited the senescent cell phenotypes and restored cell proliferation and Ki-67 expression. Sandalore also inhibited the expression of senescence-associated β-galactosidase and increased p21 expression in senescent HaCaT cells in response to hydrogen peroxide. Additionally, sandalore activated the CaMKKβ/AMPK/mTORC1/autophagy signaling axis and promoted autophagy. *OR2AT4* knockdown attenuated the increased in the intracellular calcium level, cell proliferation, and AMPK phosphorylation induced by sandalore. These findings demonstrate that the effects of sandalore are mediated by OR2AT4 activation. Our findings suggest that OR2AT4 may be a novel therapeutic target for anti-aging and anti-senescence in human keratinocytes.

## 1. Introduction

Aging is progressive physiological alterations in an organism that lead to senescence, or a decline of biological functions and of the organism’s ability in response to metabolic stress. Aging reduces health span and physiological functions primarily due to the accumulation of cellular damage, thus increasing the risk for developing chronic diseases [1].

According to the progression of aging, human skin is constantly exposed to internal and external stimuli that have an impact on its functionality, manifesting as wrinkling, dry skin, a weakened skin barrier, loss of skin integrity, epidermal thinning, and skin cancer [2,3,4]. Skin aging is regulated by multiple proteins, and it is possible that ectopic olfactory receptors (ORs) may play critical roles since the skin is constantly exposed to external odorants. ORs belong to the largest protein superfamily of guanine nucleotide-binding protein (G protein)-coupled receptors (GPCRs) and are primarily expressed in the cilia of olfactory sensory neurons. These ectopic ORs have been shown to regulate cellular and tissue-specific functions [5,6] thus aroma compounds that are ligands for ectopic ORs may have biological activities in skin.

ORs are expressed in sperm, skeletal muscles, liver, adipose tissue, colon, brain, tongue, and skin [6,7,8,9,10]. We previously found that Olfr544 activation reduces obesity by shifting the fuel preference to fat in high-fat diet-fed mice [11], by inducing mitochondrial biosynthesis in skeletal muscle [12], and by modulating glucagon-like peptide-1 secretion and intestinal inflammation [13]. These studies proved that ectopic ORs play a crucial role in various physiological processes in diverse non-olfactory tissues. Nevertheless, the physiological functions of most ectopic ORs are unknown.

ORs are expressed in several skin cells, such as melanocytes, fibroblasts, hair follicle epithelial cells, and keratinocytes [14]. Skin-expressed ORs are involved in numerous physiological activities, including wound healing [6], anti-cancer activities [15], hair growth [16], skin stress response [17], skin inflammation, barrier dysfunction [18], and atopic dermatitis [19]. Of interest, OR2AT4 is expressed in HaCaT human primary keratinocytes and is involved in wound healing and hair growth [6,20], of which activity declines with aging. Therefore, we hypothesized that OR2AT4 may regulate skin aging and senescence.

Hydrogen peroxide (H_2_O_2_), one of the most common reactive oxygen species (ROS), induces cellular senescence [21]. Intracellular H_2_O_2_ produces pro-oxidant oxygen radicals and induces oxidative stress. The ROS produced by H_2_O_2_ induces senescence and skin aging in keratinocytes. In this study, we investigated the function of OR2AT4 in senescent HaCaT keratinocytes. Cells were incubated with non-cytotoxic levels of H_2_O_2_ to produce oxidative stress and induce senescence. Then, we explored the roles of OR2AT4 in cellular aging and senescence. Our findings suggest that sandalore suppressed senescence and increased cell proliferation by activation of the calcium/calmodulin-dependent protein kinase kinase (CaMKKβ)/adenosine monophosphate-activated protein kinase (AMPK)/mammalian target of rapamycin complex 1 (mTORC1)/autophagy signaling axis.

## 2. Materials and Methods

### 2.1. Cell Culture

The HaCaT human skin keratinocyte line was purchased from the American Type Culture Collection (Manassas, VA, USA). HaCaT Cells were cultured in Dulbecco’s Modified Eagle Medium (Hyclone, Logan, UT, USA) containing 10% fetal bovine serum (Hyclone) and 1% penicillin/streptomycin (Hyclone) in an incubator at 5% CO_2_ and 37 °C.

### 2.2. Real-Time Polymerase Chain Reaction (RT-PCR) and Quantitative RT-PCR (qRT-PCR) Analyses

RT-PCR and qRT-PCR analyses were performed, as described previously [5]. Briefly, the total mRNA was extracted using RNAiso Plus (TaKaRa Bio Inc., Shiga, Japan), and the cDNA samples were obtained using ReverTra Ace qPCR RT Master Mix with a gDNA Eraser (Toyobo, Osaka, Japan), according to the manufacturer’s instructions. The RT-PCR amplification process was performed using Dreamtaq Green PCR Master Mix (Thermo Fisher Scientific, Waltham, MA, USA) and S1000 Thermal Cycler (Bio-Rad Laboratories, Hercules, CA, USA). The RT-PCR products were confirmed by agarose gel electrophoresis at a current of 5 V/cm. The images were captured using the ChemiDoc Touch System (Bio-Rad Laboratories). qRT-PCR was conducted using Thunderbird SYBR^®^ qPCR Mix reagent (TaKaRa) and was analyzed using the iQ5 Cycler System (Bio-Rad Laboratories). The mRNA expression was validated by the RT-PCR and qRT-PCR analysis of the ribosomal protein L32. The 2^−ΔΔCt^ method was used to analyze the relative changes in gene expression. The primer sequences used for the experiment are shown in Table 1.

### 2.3. Immunoblot Analysis

To detect OR2AT4, plasma membrane proteins were extracted using a plasma membrane protein extraction kit (101Bio, Palo Alto, CA, USA), according to the manufacturer’s instructions. To extract the total proteins, the cells were lysed with ice-cold RIPA buffer containing 1% halt protease and phosphatase inhibitor reagent (Thermo Fisher Scientific). The plasma membrane protein and total protein concentrations were determined using the Bradford reagent (Bio-Rad Laboratories). Equal quantities of the proteins were separated by SDS-PAGE and were transferred to nitrocellulose membranes (GVS Filter Technology, Morecambe, UK). The membranes were blocked with 5% skimmed milk for 1 h and were incubated with the primary antibody (1:1000). The primary antibodies used for immunoblotting were as follows: OR2AT4 (ab105828, 36 kDa; Abcam, Cambridge, MA, USA), GNAL (sc-48345, 46 kDa; Santa Cruz Biotechnology, Santa Cruz, CA, USA), adenylate cyclase 3 (ADCY3) (sc-588, 130 kDa; Santa Cruz Biotechnology), p21/CIP1/CDKN1A (NBP2-294563, 21 kDa; Novus, St. Louis, MO, USA), phosphorylated calcium/calmodulin-dependent protein kinase kinase (p-CaMKKβ) (12818S, 50 kDa; Cell Signaling Technology, Danvers, MA, USA), CaMKKβ (sc-50341, 50 kDa; Santa Cruz Biotechnology), phosphorylated adenosine monophosphate-activated protein kinase (p-AMPK) (44-1150G, 62 kDa; Invitrogen, Carlsbad, CA, USA), AMPK (sc-25792, 62 kDa; Santa Cruz Biotechnology), LC3 antibody (sc-0376404, 15 kDa; Santa Cruz Biotechnology), phosphorylated mammalian target of rapamycin complex 1 (p-mTORC1) antibody (ab63552, 289 kDa; Abcam), mTORC1 antibody (2983S, 289 kDa; Cell Signaling Technology), phosphorylated p70 ribosomal S6 kinase (p-p70S6K) antibody (2211S, 80 kDa; Cell Signaling Technology), p70S6K antibody (2217S, 80 kDa; Cell Signaling Technology), and β-actin (sc-47778, 43 kDa; Santa Cruz Biotechnology). The membranes were washed with Tris-buffered saline plus 0.1% Tween 20 and incubated with horseradish peroxidase-conjugated secondary mouse or anti-rabbit IgG antibody (1:5000; Invitrogen). The immunoblot images were obtained using the Chemi-Doc Touch Imaging System (Bio-Rad Laboratories). The band density was analyzed using Image Lab 5.2 software (Bio-Rad Laboratories). The protein expression was normalized to that of the β-actin expression.

### 2.4. Immunocytochemistry

The HaCaT cells were seeded in six-well confocal plates at a density of 1 × 10^6^/well. After 24 h of incubation, the cells were washed twice with phosphate-buffered saline (PBS) and fixed with 4% paraformaldehyde for 5 min at 37 °C. Then, the cells were incubated with 5% bovine serum albumin dissolved in PBS for 45 min at 37 °C. The HaCaT cells were incubated overnight with one of the following primary antibodies for immunocytochemistry: OR2AT4 (PA5-39811, 1:200; Thermo Fisher Scientific), sodium/potassium-ATPase α (Na^+^/K^+^-ATPase-α) (SC-48345, 1:50; Santa Cruz Biotechnology), and Ki-67 (NB500-170, 1:200; Novus). Then, the cells were washed three times with PBS. The HaCaT cells were incubated with anti-mouse IgG (Alexa Fluor 488; A11001, 1:500; Thermo Fisher Scientific) or anti-rabbit IgG (Alexa Fluor 532; A11009, 1:500; Thermo Fisher Scientific) secondary antibody for 1 h at room temperature. Next, the cells were incubated with 300 nM DAPI (Cayman Chemical, Ann Arbor, MI, USA) for 5 min in the dark. The cells were washed twice with PBS. Images were obtained using the LSM510 META confocal microscope and analyzed using LSM700 software (version 3.2; Carl Zeiss, Jena, Germany).

### 2.5. Cre-Luciferase Assay

For the Cre-luciferase assay, the Hana3A cells were grown in MEM containing 10% FBS with 1% PEST [22]. Hana3A cells were seeded 2 × 10^4^ cells per well in 96-well plates. The Hana3A cells were transfected with OR2AT4 (OriGene, Rockville, MD, USA) and an empty vector as the control using the DharmaFECT transfection reagent (Horizon Discovery, Cambridge, UK), and the transfection process was conducted by following the manufacturer’s guidelines. Subsequently, the control group was treated with DMSO, the positive control group was treated with 100 µM forskolin (FSK; Cayman), and the experimental group was treated with 50 and 100 µM sandalore (Givaudan Schweiz AG, Dübendorf, Switzerland) for 24 h. Luciferase activities were detected by the dual-luciferase reporter assay system (E1960, Promega, Madison, WI, USA) and VICTOR^™^ X Multilabel Plate Reader (PerkinElmer, Inc., Waltham, MA, USA).

### 2.6. cAMP Assay

For the intracellular cAMP assay, HaCaT cells were seeded in a 96-well plate at a density of 4 × 10^4^/well. After 24 h of incubation, the control group was treated with DMSO, the positive control group was treated with 100 µM forskolin, and the experimental group was treated with sandalore (50 or 100 µM) for 24 h. A screen quest fluorometric ELISA cAMP assay kit (36373; AAT Bioquest, Sunnyvale, CA, USA) and VICTOR^™^ X Multilabel Plate Reader (PerkinElmer, Inc.) were used to determine the cAMP concentration changes, according to the manufacturers’ instructions.

### 2.7. Inositol Phosphate Assay

For the intracellular inositol phosphate assay, HaCaT cells were seeded in a 384-well OptiPlate (PerkinElmer, Inc.) at a density of 4 × 10^4^/well. After overnight incubation, the control group was treated with DMSO and the experimental group was treated with 50 and 100 µM sandalore for 24 h. The inositol phosphate concentration changes were detected using the IP-One Tb kit^®^ (62IPAPEC; CisBio Bioassays, Codolet, France) and EnVision Xcite Multilabel plate reader (PerkinElmer, Inc.) equipped with homogenous time-resolved fluorescence filters. The homogenous time-resolved fluorescence ratio was calculated by dividing the raw fluorescence values measured at 665 nm by the raw signals measured at 620 nm.

### 2.8. Calcium Assay

For the intracellular calcium assay, the HaCaT cells were seeded in a 96-well plate at a density of 4 × 10^4^/well. After overnight incubation, the control group was treated with DMSO, the positive control group was treated with A23187 (10 µM; Cayman chemical), and the experimental group was treated with sandalore (100 µM, 300 µM, 1 mM, and 3 mM) for 24 h. Calcium concentration changes were detected using the fluo-4 NW calcium assay kit (F36206; Molecular Probes, Eugene, OR, USA) and VICTOR^™^ X Multilabel Plate Reader (PerkinElmer, Inc.).

### 2.9. Small Interfering RNA (siRNA) Transfection

Human OR2AT4 siRNAs were transfected using a commercial siRNA reagent system (sc-45064; Santa Cruz Biotechnology), following the manufacturer’s instructions. Briefly, the cells were transfected with 20 nM control siRNA (nontargeting siRNA) or OR2AT4 siRNA using lipofectamine 2000 (Invitrogen).

### 2.10. Induction of Senescence in HaCaT Cells

For the induction of premature senescence, For the induction of premature senescence, HaCaT cells were treated with 200 µM H_2_O_2_ (Sigma-Aldrich, St. Louis, MO, USA) for 3 days. The cells were pretreated with 50 or 100 µM sandalore for 1 h before the addition of H_2_O_2_. The positive control was treated with 25 µM quercetin (Sigma-Aldrich).

### 2.11. Senescence-Associated β-Galactosidase (SA-β-gal) Staining

SA-β-gal staining was performed according to a previously published protocol [23]. At pH 6.0, positive staining of β-galactosidase has been reported to remarkably increase in senescent cells. Briefly, the cells were washed twice with PBS and fixed with 0.2% glutaraldehyde for 15 min at room temperature. The fixed cells were washed with PBS and incubated with a freshly prepared SA-β-gal staining solution (1 mg/mL X-gal, 5 mM K_3_FeCN_6_, 5 mM K_4_FeCN_6_, and 2 mM MgCl_2_) overnight at 37 ℃ without CO_2_. The positive blue-stained cells were counted under a microscope at 100× magnification and expressed as a percentage using ImageJ software (National Institutes of Health, Bethesda, MD, USA).

### 2.12. Cell Counting and Proliferation Assay

The HaCaT cells were treated with DMSO as a control, quercetin (25 µM), and sandalore (50 and 100 µM) for 1 h, and then co-treated with 200 µM H_2_O_2_ for 72 h. The cells were detached by trypsinization for counting. The total viable cells were counted using the automated cell counter Adam-MC with the Adam MC AccuChip 4× kit (NanoEnTek Inc., Seoul, Korea). Cell proliferation was measured using the CyQUANT^®^ NF Cell Proliferation Assay Kit (Invitrogen), according to the manufacturer’s instructions. Cell proliferation was measured based on fluorescence using the VICTOR^TM^ X Multilabel Plate Reader (PerkinElmer) at an excitation wavelength of 485 nm and emission wavelength of 535 nm.

### 2.13. Monodansylcadaverine (MDC) Staining

For MDC staining, the cells were treated with 50 µM MDC (Sigma-Aldrich) in the medium and were incubated at 37 °C for 20 min. The MDC-stained autophagic vacuoles were examined by fluorescence microscopy. Images were obtained using the LSM510 META confocal microscope and were analyzed using LSM700 software (version 3.2; Carl Zeiss, Jena, Germany). The MDC-stained cells were counted using ImageJ software (National Institutes of Health).

### 2.14. Statistical Analysis

Data are expressed as means ± standard error of the mean (SEM) based on at least triplicate experiments. Student’s *t*-test was used to compare the groups. Statistical analysis was performed using GraphPad Prism 8 (GraphPad Software Inc., San Diego, CA, USA). A *p*-value < 0.05 was considered statistically significant.

## 3. Results

### 3.1. OR2AT4 and OR Signaling Components Are Expressed in Human Keratinocytes

**Figure 1:**
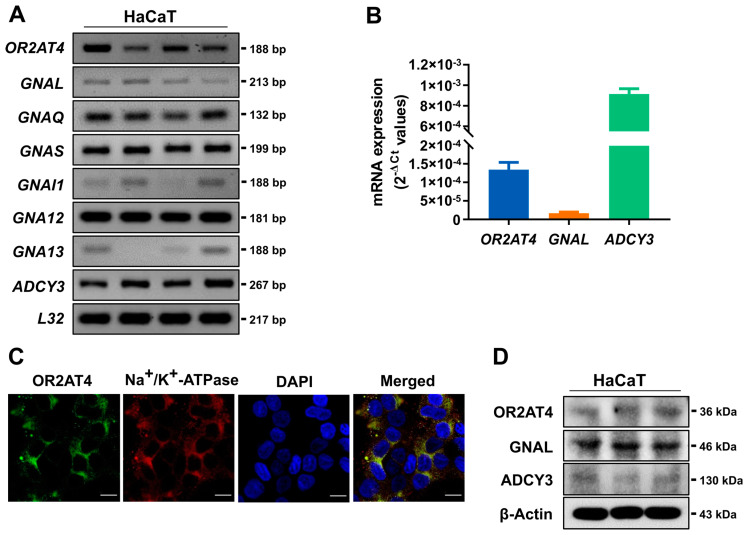
Ectopic expression of OR2AT4 in HaCaT human keratinocytes. (**A**) mRNA expression of *OR2AT4*, *GNAL*, and *ADCY3* in HaCaT cells by RT-PCR. Experiments were done in tetraplicate. (**B**) The mRNA expression of *OR2AT4*, *GNAL*, and *ADCY3* in HaCaT cells by real-time qPCR. (**C**) Representative images of immunocytochemical staining of OR2AT4. OR2AT4 (red) and Na^+^/K^+^-ATPase, a plasma membrane marker (green): the merged image (yellow) shows colocalization of Na^+^/K^+^-ATPase and OR2AT4 in the plasma membrane. Scale bar, 20 µm. (**D**) Immunoblot analysis of the membrane fraction of HaCaT cells. Experiments were done in triplicate. Quantification data are means ± standard error of mean (SEM).

OR2AT4 is ectopically expressed in human primary keratinocytes, and sandalore is a ligand of OR2AT4 [6]. We confirmed the expression of OR2AT4 and its signaling components in HaCaT human keratinocytes (Figure 1A,B). G proteins play crucial roles in signal transmission via membrane-bound receptors along cell membranes [24,25]. The RT-PCR and RT-qPCR analyses showed that *OR2AT4*, *GNAL*, and *ADCY3* are co-expressed in HaCaT cells (Figure 1A,B). GNAL is a G protein isoform that interacts with ORs, and ADCY3 produces cAMP as an effector of GNAL [24]. The RT-PCR results demonstrated that *GNAQ*, *GNAS*, *GNAI1*, *GNAI1*, *GNA12*, and *GNA13* were also expressed in HaCaT cells. Immunocytochemistry demonstrated that OR2AT4 is colocalized with Na^+^/K^+^-ATPase, a cell surface marker (Figure 1C). Immunoblotting revealed that OR2AT4, GNAL, and ADCY3 proteins are expressed in the membrane fraction of HaCaT cells (Figure 1D). These results suggest that OR2AT4 is expressed on the surface membrane of OR2AT4 together with OR signaling components.

### 3.2. Activation of OR2AT4 by Sandalore in Human Keratinocytes

**Figure 2:**
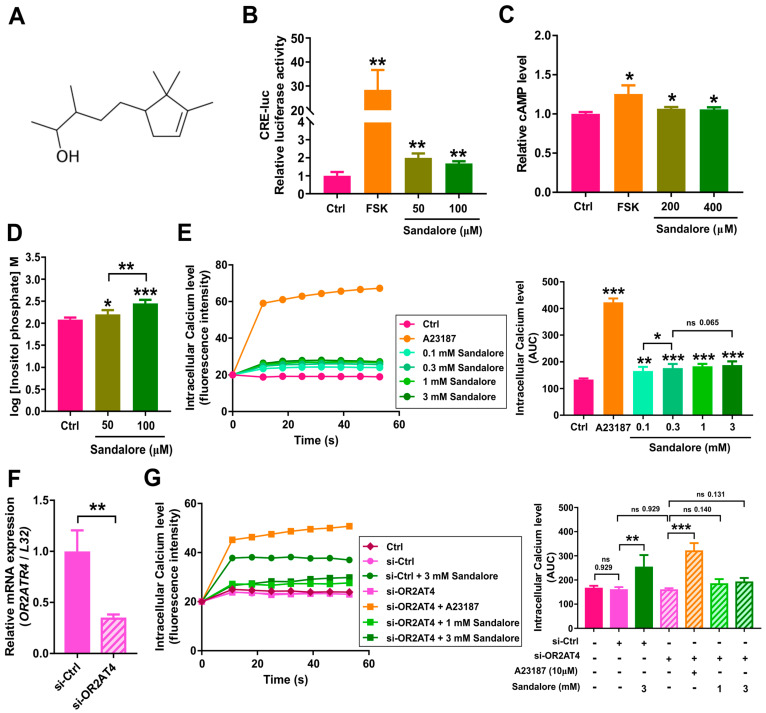
Second messenger analysis and CRE activity measurements in HaCaT cells treated with sandalore. (**A**) Chemical structure of sandalore. (**B**) CRE-luciferase activity in Hana3A cells transfected with OR2AT4. (**C**) Relative cAMP, (**D**) inositol phosphate, and (**E**) intracellular calcium levels in HaCaT cells treated with sandalore. (**F**) *OR2AT4* mRNA expression in cells transfected with siRNAs. si-Ctrl, HaCaT cells transfected with control siRNA; si-OR2AT4, HaCaT cells transfected with OR2AT4 siRNA. (**G**) Intracellular calcium levels in HaCaT cells with *OR2AT4* knockdown. Ctrl, control; FSK, 1 µM forskolin; A23187, 10 µM calcium-ionophore; AUC, area under curve. Data are means ± SEM. * *p* < 0.05, ** *p* < 0.01, and *** *p* < 0.001. Student’s *t*-test was performed for comparisons between groups.

Next, we investigated the second messengers induced by sandalore in HaCaT cells. Sandalore consists of a 2,2,3-trimethylcyclopent-3-enyl structure with a polar OH group (Figure 2A) and is a ligand for OR2AT4 [6]. In the CRE-luciferase assay using OR2AT4-transfected Hana3A cells [22], sandalore significantly activated OR2AT4 (Figure 2B). In the second messenger analysis, sandalore treatment induced all three second messengers, i.e., cAMP (Figure 2C), inositol phosphate (Figure 2D), and calcium (Figure 2E), in HaCaT cells. To determine whether the sandalore effect depends on OR2AT4, we knocked down the *OR2AT4* gene. The mRNA expression of OR2AT4 was reduced by approximately 70% in the cells transfected with OR2AT4 siRNA (Figure 2F). The increase in the calcium level in HaCaT cells by sandalore was attenuated by *OR2AT4* knockdown (Figure 2G). Our results indicate that OR2AT4 activation by sandalore stimulates complex secondary messenger responses, and that the increase in the intracellular calcium level depends on OR2AT4.

### 3.3. OR2AT4 Activation by Sandalore Recovers the Proliferation Capacity of H_2_O_2_-Induced Senescent HaCaT Cells

**Figure 3:**
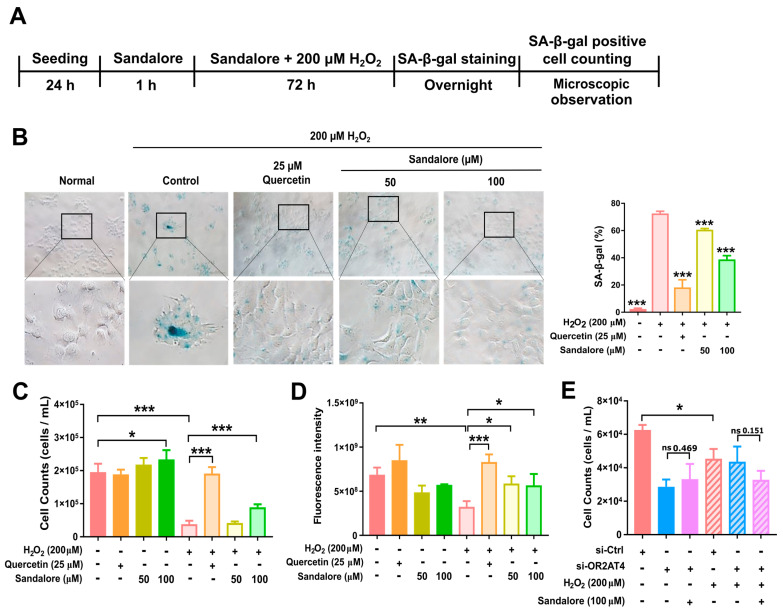
OR2AT4 activation by sandalore restored the senescence phenotype and cell proliferation in HaCaT cells. (**A**) Schematic representation of the experimental design. (**B**) Representative images of SA-β-gal staining. Original magnification, 200×. Scale bar, 50 µm (left panel). Quantification of SA-β-gal-positive cells (right panel) was analyzed using ImageJ software. (**C**) Live cell counting (*n* = 6), (**D**) cell proliferation assay (*n* = 3), and (**E**) live cell counting in *OR2AT4*-knockdown HaCaT cells. Quercetin was used as a positive control. Data are means ± SEM. * *p* < 0.05, ** *p* < 0.01, and *** *p* < 0.001. Student’s *t*-test was performed for the comparisons between groups.

Aging is a complex biological process characterized by cellular senescence. To understand the role of OR2AT4 in the aging of human keratinocytes, we investigated whether OR2AT4 regulates cellular senescence in HaCaT cells. H_2_O_2_ induces cellular senescence, mainly by inducing oxidative stress. Senescence induced by H_2_O_2_ is termed stress-induced premature senescence [21,26,27]. Senescence was induced in HaCaT cells using different concentrations of H_2_O_2_ and incubation times. The results showed that treatment with 200 μM H_2_O_2_ for 72 h induced senescence in HaCaT cells with minimal apoptosis and cell death. Additionally, HaCaT cells were treated with sandalore for 1 h prior to co-treatment with sandalore and H_2_O_2_ for 72 h (Figure 3A). After the induction of senescence by H_2_O_2_, the SA-β-gal expression was evident in HaCaT cells (Figure 3B). H_2_O_2_-treated HaCaT cells were enlarged, flattened, and irregularly shaped, which are characteristics of senescent cells, suggesting that HaCaT cells underwent senescence (Figure 3B; left panel). The quantification of SA-β-gal expression showed that the proportion of SA-β-gal positive cells was significantly higher in senescent cells compared with cells that were not treated with H_2_O_2_, whereas sandalore significantly decreased the proportion of SA-β-gal-positive cells (Figure 3B, right panel). These results demonstrate that sandalore effectively reduced senescence in HaCaT keratinocytes. Next, we examined the effect of sandalore on cell proliferation. The viable cell counting and CyQUANT cell proliferation assays showed that sandalore significantly increased proliferation in the H_2_O_2_-induced senescent cells (Figure 3C,D). The effects of sandalore were attenuated in senescent cells with *OR2AT4* knockdown (Figure 3E). These results indicate that sandalore recovers the proliferation capacity of senescent cells in an OR2AT4-dependent manner.

**Figure 4:**
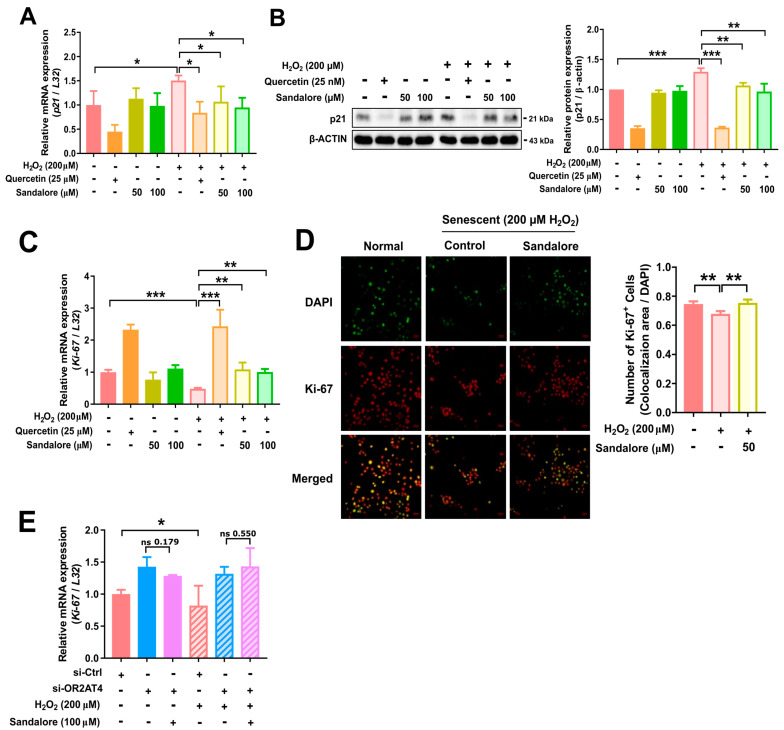
Sandalore suppressed senescence as assessed by the expression of p21 and Ki-67. (**A**) The mRNA expression of *p21* in non-senescent and senescent cells. (**B**) Immunoblot analysis of p21. The relative protein expression of p21 was analyzed using ImageLab software. (**C**) Relative mRNA expression of *Ki-67*. (**D**) Representative images of immunocytochemical staining in HaCaT cells. Immunolocalization of Ki-67 (red) and DAPI (green): the merged image (yellow) shows colocalization of DAPI and Ki-67 in HaCaT cells. DAPI staining was imaged using correctly adjusted acquisition settings and was displayed in a green pseudo color. Scale bar, 50 µm (left panel). Ki-67-positive HaCaT cells (right panel) were quantified using ImageJ software. (**E**) Relative mRNA expression of *Ki-67* in *OR2AT4*-knockdown HaCaT cells. Data are means ± SEM. * *p* < 0.05, ** *p* < 0.01, and *** *p* < 0.001. Student’s *t*-test was performed for comparisons between groups.

Next, we assessed the expression of p21 and Ki-67. p21 is a pleiotropic inhibitor of the cyclin/cyclin-dependent kinase complexes that mediate cell cycle progression. The induction of p21 causes cell cycle arrest in senescent cells [28]. Ki-67 is a nuclear protein that is highly expressed in asynchronously cycling cells and is absent in non-dividing cells [29]. It is a common marker of proliferating cells [30]. The H_2_O_2_-induced senescent cells expressed a higher expression of *p21* mRNA compared with non-senescent cells (Figure 4A). Quercetin, a positive control, reduced the *p21* mRNA expression in non-senescent and senescent cells. Sandalore did not affect the *p21* expression in non-senescent cells, but significantly suppressed the *p21* expression in senescent cells (Figure 4A). The immunoblotting analysis showed similar trends for p21 protein and mRNA expression (Figure 4B).

The *Ki-67* mRNA expression was lower in senescent cells than non-senescent cells (Figure 4C). Sandalore significantly increased the *Ki-67* gene expression in senescent cells. Additionally, quercetin increased the *Ki-67* mRNA expression in non-senescent and senescent cells. Sandalore selectively increased the *Ki-67* expression in senescent cells, but not non-senescent cells (Figure 4C). These findings suggest that sandalore may recover the proliferation of senescent cells but not non-senescent cells, which is in line with the p21 expression results (Figure 4A,B). The immunocytochemical analysis showed that the Ki-67 expression was significantly increased in senescent HaCaT cells. Ki-67-positive cells were significantly increased in the cells treated with sandalore compared with the control senescent cells (*p* < 0.01; Figure 4D). Importantly, the sandalore induction of the *Ki-67* mRNA expression in HaCaT cells was attenuated by *OR2AT4* knockdown (Figure 4E). Taken together, our results demonstrate that sandalore treatment recovers cell proliferation by regulating p21 and Ki-67 in HaCaT cells via OR2AT4-dependent mechanisms.

### 3.4. OR2AT4 Activation by Sandalore Stimulates the CaMKKβ/AMPK/mTORC1 Signaling Pathway to Suppress Senescence in HaCaT Cells

**Figure 5:**
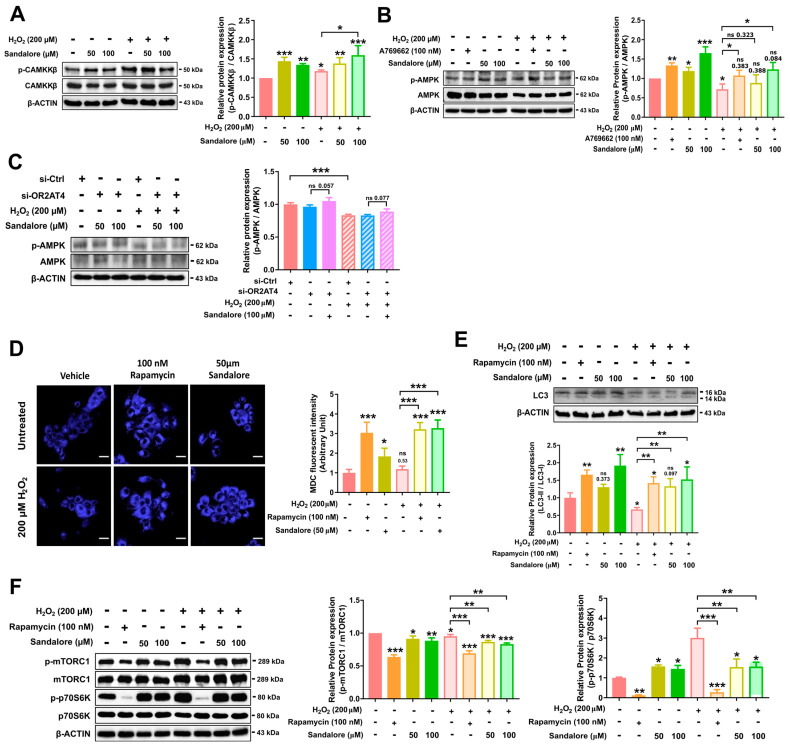
OR2AT4 activation by sandalore mediates the CAMKKβ/AMPK/mTORC1/autophagy axis. Expression of total and phosphorylated (**A**) CAMKKβ and (**B**) AMPK by immunoblot analysis. (**C**) Representative images of MDC-stained HaCaT cells. Scale bar, 20 µm (left panel). Quantification of the MDC fluorescence intensity in HaCaT cells (right panel). Fluorescence was quantified using the VICTOR^™^ X Multilabel Plate Reader. (**D**) The expression of LC3I and LC3II by immunoblot analysis (left panel). Bar graphs represent the ratio of the LC3II to LC3I within each sample (right panel). (**E**) Expression of the total and phosphorylated mTORC1 and 70S6K by immunoblot analysis. (**F**) Expression of the total and phosphorylated AMPK in the *OR2AT4*-knockdown HaCaT cells. The relative protein expression of p-CAMKKβ/CAMMKβ, p-AMPK/AMPK, p-mTORC1/TORC1, and p-p70S6K/p70S6K were analyzed using ImageLab software. Data are means ± SEM. * *p* < 0.05, ** *p* <0.01, and *** *p* < 0.001. Student’s *t*-test was performed for comparisons between groups.

To explore the mechanisms underlying the suppression of senescence by OR2AT4, we investigated the cellular signaling pathway regulated by sandalore-induced OR2AT4 activation in HaCaT cells. Sandalore increased the intracellular calcium concentration in an OR2AT4-dependent manner. We performed immunoblotting to evaluate CaMKKβ phosphorylation, which is activated by intracellular calcium. Cytosolic calcium stimulates the CaMKKβ signaling cascade, leading to several cellular responses [31,32]. Furthermore, activated CaMKKβ phosphorylates AMPK [32]. Sandalore increased the phosphorylation of CaMKKβ and AMPK in both non-senescent and senescent cells (Figure 5A,B). In *OR2AT4*-knockdown cells, the effect of sandalore on AMPK phosphorylation was attenuated (Figure 5C). Our results demonstrated that sandalore increased the phosphorylation of CaMKKβ and its downstream target AMPK in an OR2AT4-dependent manner (Figure 5A,B).

AMPK activation stimulates autophagy [33,34], which can be assessed in vitro by MDC staining [35]. Thus, we evaluated the induction of autophagy by sandalore using MDC, an auto-fluorescent dye, in HaCaT cells. We found that sandalore increased autophagy in senescent HaCaT cells (Figure 5C). During autolysosome formation, LC3II is processed from the cytosolic form (LC3I) to the membrane-bound form (LC3II), thus the LC3II/LC3I ratio is a key molecular marker of autophagy [36]. In the immunoblot analysis, the induction of senescence by H_2_O_2_ reduced the LC3II/LC3I ratio compared with that in non-senescent cells, suggesting the inhibition of autophagy in senescent cells. Rapamycin, a known autophagy inducer, increased the LC3II/LC3I ratio in non-senescent and senescent cells. OR2AT4 activation by sandalore (100 μM) also increased the LC3II/LC3I ratio in non-senescent and senescent cells (Figure 5D). These findings suggest that sandalore activates cellular autophagy in non-senescent and senescent cells.

One of the major mechanisms of AMPK-dependent autophagy activation is the inhibition of mTORC1 phosphorylation. In the immunoblot analysis, the senescent cells showed increased phosphorylation of mTORC1 and its target protein p70S6K compared with the non-senescent cells (Figure 5D). Rapamycin and sandalore decreased the phosphorylation of mTORC1 and p70S6K (Figure 5E). These results demonstrate that OR2AT4 activation by sandalore stimulates the CaMKKβ/AMPK/mTORC1/autophagy signaling axis. It has been reported that the activation of the CaMKKβ/AMPK/mTORC1 signaling axis can suppress aging and senescence in multiple cells and tissues [37,38].

## 4. Discussion

The skin is the largest organ and forms a mechanical barrier that senses numerous external chemicals via the cell surface sensory receptors, such as ectopic ORs [39,40]. Ectopic ORs play significant roles in several tissues, including keratinocytes and other skin cells [6,16,41]. For example, the function of OR2AT4 has been evaluated in skin cells including keratinocytes [6,16,42]. OR2AT4 activation increased human keratinocyte proliferation, migration, and regeneration in vitro, as well as wound re-epithelialization and hair growth ex vivo [6]. OR2AT4 expression in keratinocytes promotes wound healing and hair growth, of which the activity declines with aging. Therefore, we hypothesized that OR2AT4 may suppress aging and senescence in keratinocytes. In the present study, we investigated whether OR2AT4 could regulate cellular aging and senescence in human keratinocytes. To understand the functions of OR2AT4 in aging and the underlying mechanisms, we added sandalore, a ligand of OR2AT4, to H_2_O_2_-induced premature senescent HaCaT human keratinocytes. First, we confirmed the expression of OR2AT4 and OR signaling components, including GNAL and ADCY3, in HaCaT cells. Calcium plays important roles in regulating the sensitivity of olfactory transduction as part of odor adaptation, because odorant adaptation involves modulation of the cAMP-gated channel by calcium feedback [43]. OR2AT4 activation by sandalore increased the intracellular calcium, cAMP, and inositol phosphate levels, whereas *OR2AT4* knockdown attenuated the increased calcium level, suggesting that sandalore increased the calcium level via OR2AT4 activation.

Human skin senescence is a progressive process that involves intrinsic or extrinsic aging [39]; increased susceptibility to wrinkles; and decreased self-healing properties, dermal elasticity, and epidermal barrier maintenance [2,3,4]. Skin senescence, which is mediated by time and environmental factors, proceeds via a common molecular pathway that involves ROS formation [3,4,44,45]. In human cells, H_2_O_2_ is naturally produced during many physiologic and pathological processes. It is commonly used as a model pro-oxidant to evaluate oxidative stress and premature senescence [21,27]. Stress-induced premature senescence in response to H_2_O_2_ exposure involves different pathways than those of replicative senescence [21,26,27]. In our study, OR2AT4 activation ameliorated SA β-galactosidase activity and increased cell proliferation and Ki-67 expression, a marker of cell proliferation. Quercetin treatment was also associated with significantly increased cell proliferation in non-senescent and senescent cells; however, sandalore increased cell proliferation in senescent cells, but not in non-senescent cells. These findings suggest that sandalore and OR2AT4 may recover cell proliferation activity particularly in senescent cells, without affecting normal cells, and may be beneficial for clinical applications. Our results demonstrate that OR2AT4 activation by sandalore suppresses the expression of p21, a senescence-associated secretory phenotype factor and cell cycle arrest factor, in senescent cells. p21 is involved in cell cycle arrest in the G1 phase of senescent cells [46]. Additionally, p21 activates P53-induced cell cycle arrest or apoptosis [47]. Cellular senescence and cell cycle arrest involve essential checkpoints of cellular homeostasis, such as the maintenance of DNA replication, repair, and division [48,49]. Increased cell proliferation by sandalore in senescent cells was attenuated by *OR2AT4* knockdown. Therefore, increased cell proliferation and Ki67 expression, as well as decreased SA-β gal and p21 expression, in senescent cells demonstrated that sandalore may recover the cell proliferation capacity of senescent cells via an OR2AT4-dependent mechanism.

Calcium, a key second messenger, activates diverse signal transduction pathways, including the calmodulin kinase pathway. Calmodulin kinase, a Ser/Thr kinase, phosphorylates the Thr residue in the activation loop of CaMKKβ, which further activates AMPK by phosphorylating the ^172^Thr of the alpha subunit. AMPK is a critical regulator of cellular energy metabolism, proliferation, and autophagy. Intracellular calcium signaling activates AMPK and autophagy [34]. In the present study, sandalore increased the phosphorylation of CaMKKβ and AMPK, which was suppressed by senescence. The increased AMPK phosphorylation by sandalore was inhibited in cells with *OR2AT4* knockdown. These findings suggest that OR2AT4 activation by sandalore enhanced the calcium/CaMKKβ/AMPK signaling axis.

The mTORC1 signaling pathway is one of the major pathways that increases autophagy. AMPK inhibits mTORC1 directly via the phosphorylation of raptor, a component of the mTORC1 complex, and via the phosphorylation of TSC1/2, an inhibitor of mTORC1 [50]. Inhibition of mTORC1 reduces protein synthesis and promotes autophagy [33].

Our results suggest that sandalore decreased the phosphorylation of mTORC1 and P70S6K, a downstream target of mTORC1, in the H_2_O_2_-induced senescence of human keratinocytes. Autophagosome formation was markedly increased in H_2_O_2_-induced senescent keratinocytes via OR2AT4 activation by sandalore treatment. The autophagy content was measured by both MDC staining and immunoblotting of the LC3II/LC3I ratio, respectively. Both results consistently demonstrated that autophagy was increased through the activation of OR2AT4 by sandalore. Autophagy is generally reduced in cells under pathological conditions, such as aging [51]. Therefore, OR2AT4 activation by sandalore increased autophagosome formation, at least partly, via the calcium/CaMKKβ/AMPK/mTORC1 signaling axis.

mTORC1 signaling increases the senescence-associated secretory phenotype gene expression, and the expression of p21 is increased by the mTORC1/P70S6K pathway [52,53]. Our results showed that the p21 expression is reduced by sandalore in senescent cells, suggesting that sandalore suppressed the p21 expression by inhibiting the mTORC1/P70S6K pathway via AMPK activation in senescent cells. On the other hand, P70S6K phosphorylation was increased by sandalore in non-senescent cells, while the upstream kinase mTORC1 was suppressed. The reasons for the unexpected results are unclear. It is possible that sandalore may have marginal effects on normal non-senescent cells or have additional direct effects on P79S6K in an mTORC1-independent manner.

## 5. Conclusions

**Figure 6:**
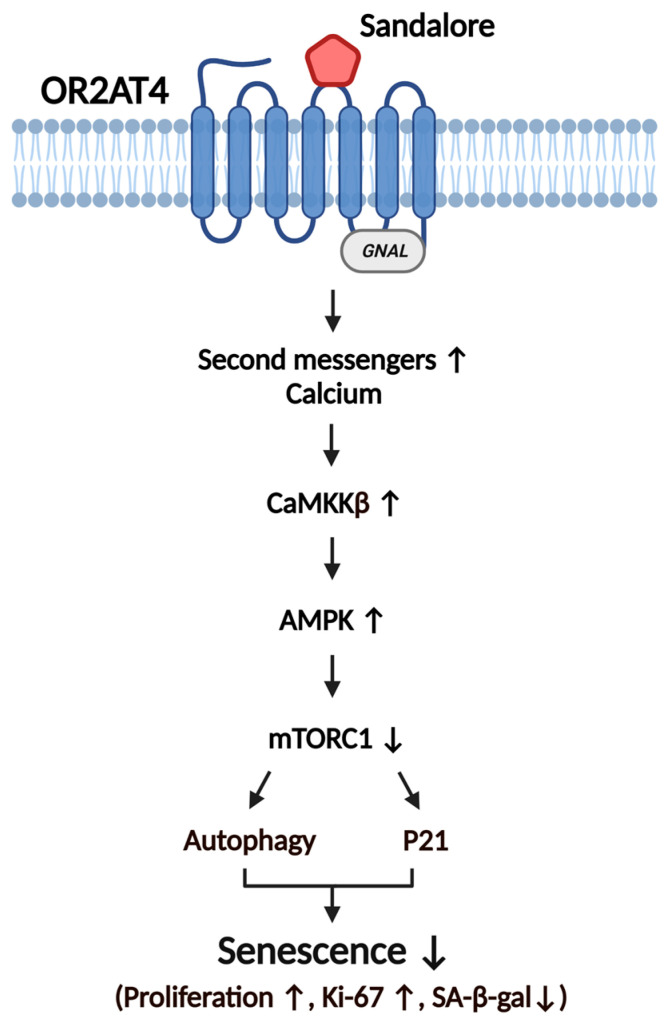
Proposed role of OR2AT4 in the suppression of senescence in human keratinocytes. Sandalore binds and activates OR2AT4 to increase the levels of second messengers, mainly intracellular calcium. The increased intracellular calcium level activates the CAMKKβ/AMPK/mTORC1/autophagy signaling axis. OR2AT4 inhibits p21, a marker of cellular senescence, by activating AMPK phosphorylation. Overall, OR2AT4 activation by sandalore may improve cellular senescence in human keratinocytes.

This is the first study to demonstrate that OR2AT4 activation by sandalore inhibits H_2_O_2_-induced senescence of human keratinocytes. OR2AT4 activation by sandalore induced the secondary messengers, including calcium, phosphorylated AMPK, and cell proliferation in senescent cells, of which the effects were negated in the gene knockdown experiments. The suppression of senescence by OR2AT4 activation was confirmed by evaluating the expression of several senescence markers, including SA-β-gal staining, cell proliferation rate, Ki67, and p21, in H_2_O_2_-induced senescent keratinocytes. OR2AT4 activation by sandalore restored cell proliferation via activation of the CaMKKβ/AMPK/mTORC1/autophagy signaling axis (Figure 6). Our results suggest that OR2AT4 may be a possible therapeutic target for suppressing the aging and senescence of keratinocytes.

## Figures and Tables

**Table 1:** Primer sequences used for real-time quantitative PCR.

Genes ^1^	Accession No.	Forward (5′-3′)	Reverse (5′-3′)
*OR2AT4*	NM_001005285.2	CACTGTCCCCAAGATGCTGT	GTGGGTTCATGAGGACAGGG
*GNAL*	NM_182978.4	GGCCAACCCTGAAAACCAAT	GGTCTGTGGGTGTGTAGTCA
*GNAS*	NM_001410913.1	TGCTTCAACGATGTGACTGC	CAAGGACTTTCTCAGCGAGC
*GNAQ*	NM_002072.5	GCCACTGGCCTCCTATTGTT	TGCTTGCCTTGGTATGCTGA
*GNAI1*	NM_002069.6	AAGGGTGCCACATGGTGTAG	TAGTGGCTGCTGTGTCTGTG
*GNA12*	NM_007353.3	CACCCTTGGCTTGTTTTCCG	CAAGCCCAGCAAACACTGAC
*GNA13*	NM_001282425.2	AGGGAACTTTTTGCCCGAGA	ACCCTCATACCTGACCGTGA
*ADCY3*	NM_001320613.2	TCAAAACCATTGGCAGCACG	TGTCGTAGTGTGGTTTCCGG
*P21*	NM_000389.5	ACTTCCTCCTCCCCACTTGT	CACCCTGCCCAACCTTAGAG
*Ki-67*	NM_002417.5	GCCCCTAAAGTAGAACCCGT	GGGTTCGGATGATTTGCCTC
*L32*	NM_000994.4	AGAAGTTCATCCGGCACCAG	CACTTCCAGCTCCTTGACGT

^1^*OR2AT4*, olfactory receptor 2AT4; *GNAL*, guanine nucleotide-binding protein (G protein) G(olf) subunit α; *GNAS*, G protein G(s) subunit α; *GNAQ*, G protein G(q) subunit α; *GNAI1*, G protein G(i) subunit α-1; *GNA12*, G protein subunit α-12; *GNA13*, G protein subunit α-13; *ADCY3*, adenylate cyclase 3; *L32*, ribosomal protein L32.

## Data Availability

The data presented in this study are available in article.
